# Mechanisms of Action and Biomarker Potential of ncRNAs in Hepatocellular Carcinoma and Liver Fibrosis: A Review

**DOI:** 10.1155/ancp/2283163

**Published:** 2026-02-18

**Authors:** Eun Jung Sohn, Sae-Ock Oh

**Affiliations:** ^1^ Department of Biotechnology, Inje University, 197 Inje-ro, Gimhae, Gyeongsangnam-do, Republic of Korea, inje.ac.kr; ^2^ Dongguk University-WISE, Gyeongju, 38066, Gyeongsangbuk-do, Republic of Korea, dongguk.edu; ^3^ College of Natural Science, Changwon National University, Changwon, Gyeongsangnam-do, Republic of Korea, changwon.ac.kr; ^4^ Department of SmartBio, Kyungsung University, Busan, 48434, Republic of Korea, ks.ac.kr; ^5^ Research Center for Molecular Control of Cancer Cell Diversity, Pusan National University, Yangsan, Gyeongsangnam-do, Republic of Korea, pusan.ac.kr; ^6^ Department of Anatomy, School of Medicine, Pusan National University, Yangsan, Gyeongsangnam-do, Republic of Korea, pusan.ac.kr

## Abstract

Liver diseases constitute a major global health burden, contributing substantially to morbidity and mortality worldwide. Although effective therapeutic strategies have been developed for key risk factors, such as hepatitis C infection, therapeutic options for most other liver diseases remain limited. Noncoding RNAs (ncRNAs), which regulate diverse cellular and molecular processes, have emerged as promising therapeutic targets and biomarkers in liver diseases. In this study, we summarize the mechanisms of action and functional roles of ncRNAs, such as long ncRNAs (lncRNAs), circular RNAs (circRNAs), and PIWI–interacting RNAs (piRNAs), to improve therapeutic strategies and diagnostic precision in liver diseases, such as liver fibrosis and hepatocellular carcinoma (HCC).

## 1. Introduction

Liver diseases, such as chronic viral hepatitis, nonalcoholic fatty liver disease (NAFLD), and alcoholic liver disease, are common causes of death, particularly in developing countries [1]. Additionally, hepatocellular carcinoma (HCC), the predominant form of primary liver cancer, is a fatal disease with a high global prevalence rate, which is not only increasing in developing counties but also in the United States and Europe [1]. NAFLD, alcoholism, viral hepatitis B and C, diabetes mellitus, and exposure to dietary aflatoxins are major risk factors for HCC [2]. Most HCCs occur in chronic hepatitis and cirrhosis. For patients with inoperable HCC, practical therapeutic options remain limited. Although agents, such as sorafenib and immune checkpoint inhibitors, have been evaluated, their proven clinical efficacy is modest. Thus, further research is needed to identify and develop novel therapeutic approaches [3].

Hepatic fibrogenesis is a major determining factor in the onset and progression of chronic liver disease [3]. It is characterized by a chronic injury‐induced wound‐healing response in the liver, which is accompanied by excessive accumulation of extracellular matrix (ECM). This can result in the formation of fibrous scars that replace hepatocytes, thereby altering the structure of liver tissues [4]. Activated hepatic stellate cells (HSCs) are the principal ECM–producing cells in chronic liver fibrogenesis and transdifferentiate into myofibroblast‐like cells that drive excessive ECM deposition [5]. Inflammatory responses associated with liver fibrogenesis further stimulate various fibrogenic processes in HSCs [6].

Noncoding RNAs (ncRNAs) play important roles in several liver diseases. Based on a cutoff size of 200 nucleotides, regulatory ncRNAs are categorized into small ncRNAs (sncRNAs) and long ncRNAs (lncRNAs) [7]. Specifically, sncRNAs include PIWI‐interacting RNAs (piRNAs), microRNAs (miRNAs), and small interfering RNAs (siRNAs) [8]. These sncRNAs primarily regulate gene transcription and translation [9] and play a variety of structural and functional roles in RNA splicing, chromatin structure determination, and epigenetics [10]. Hence, sncRNAs are involved in the pathogenesis of various human diseases.

miRNAs are small, noncoding, typically 19–25‐nucleotide‐long RNA molecules that play crucial roles in posttranscriptional gene regulation by binding messenger RNA (mRNA) molecules and inducing mRNA degradation or translational repression [11]. In the context of liver diseases, miRNAs have been extensively studied and are recognized as key regulators of various physiological and pathological processes, including liver development, inflammation, fibrosis, metabolism, and carcinogenesis [12, 13].

lncRNAs lack open reading frames, can respond to various stimuli, and accurately reflect the actions of transcription factors or signaling pathways [7]. They also function as scaffolds, assembling proteins to form ribonucleoprotein complexes. Furthermore, they play crucial roles in various biological activities and maintain precise control over intermolecular interactions and signaling events [14]. lncRNAs can also influence cancer epigenomes, cellular processes, and pathological conditions. Long intergenic ncRNAs (lincRNAs) are a subset of lncRNA loci that do not overlap with protein‐coding genes [15].

Circular RNAs (circRNAs) are critical regulators of various biological processes [16, 17]. Most circRNAs are generated through pre‐mRNA back‐splicing, which involves the linking of a downstream 5^′^ splice site with an upstream 3^′^ splice site [18, 19]. circRNAs exert diverse functions, including acting as miRNA sponges, associating with RNA‐binding proteins, encoding proteins, and coordinating transcription processes [20].

piRNAs are a class of sncRNAs that are 26–31 nucleotides in length and are the least expressed of all sncRNA classes in animal cells [21]. They form functional RNA–protein complexes by interacting with the PIWI subfamily of Argonaute proteins [22]. Specifically, piRNAs bind to PIWI proteins to form PIWI/piRNA complexes, which recognize and bind transposons, cleave mRNA produced by transposon gene transcription, or repress transposon genes in cell nuclei. Thus, piRNAs regulate germ cell self‐renewal and stem cell proliferation and differentiation [23, 24]. In addition to their expression in mammalian germ lines, piRNAs are expressed in various human organs in a tissue‐specific manner. Furthermore, piRNAs modulate important signaling pathways at the transcriptional and posttranscriptional levels [25].

Several studies have demonstrated the diagnostic value of ncRNAs, including circulating and serum ncRNAs, in liver diseases [26]. In this review, we summarize the mechanisms of action of lncRNAs, circRNAs, and piRNAs in liver diseases and explore their potential as biomarkers. Table 1 provides details on aberrantly expressed ncRNAs in liver diseases and their molecular targets. Additionally, we highlight the most recent advancements in understanding the role of ncRNAs in the etiology of HCC and liver fibrosis, with the goal of facilitating the development of new therapeutic approaches for liver diseases.

**Table 1:** Noncoding RNAs expressed in liver diseases and their molecular targets.

Noncoding RNAs		Expression status in liver diseases	Molecular target	Reference
lncRNA	lincRNA‐00974	Upregulated in HCC	KRT19	[27]
	lncRNA PVT1	Upregulated in HCC tissue	miRNA‐214	[28]
	lincRNA‐p21	Upregulated during liver fibrosis	miRNA‐30	[29]
	lincRNA‐p21	Downregulated in HCC tissues	miRNA9/cadherin	[30]
	lncRNA URHC	Upregulated in HCC tissues	ZAK/ERK/MAPK	[31]
circRNA	circMTO1	Downregulated in HCC tissues	miRNA‐9 and miRNA‐541‐5p	[32, 33]
	circ100338	Upregulated in HCC tissues	miRNA‐141‐3p	[34]
	circLARP4	Decreased expression in patients with HCC	miRNA‐761	[29]
	circBIRC6	Upregulated in HCC tissues	miRNA‐3918	[35]
	circCDR1as	Upregulated in HCC tissues	miRNA‐1287	[36]
	circ0078710	Upregulated in HCC tissues	miRNA‐31	[37]
	circFBXW4	Downregulated during liver fibrogenesis	miRNA‐18b‐3p	[38]
	circ608	Downregulated in NASH‐related liver fibrosis	miRNA‐222	[39]
	circSMARCA5	Downregulated in iCCA and HCC tissues	None	[40]
	circ0001649	Downregulated in CCA and HCC	miRNA‐127‐5p/miRNA‐612/miRNA‐4688	[41, 42]
	circ0004018	Downregulated in HCC	miRNA‐626/DKK3	[43]
	circ0005230	Upregulated in CCA	miRNA‐1238 and miRNA‐1299	[44]
	circACTN4	Upregulated in iCCA tissues	miRNA‐424‐5p	[45]
	circNFIB (hsa_circ0086376)	Downregulated in iCCA tissues	MAPK1/ERK signaling	[46]
	circ0059961	Downregulated in iCCA tissues	miRNA‐629‐5p/SFRP2	[47]
piRNA	piR823	Upregulated in liver cirrhosis and HCC	α‐SMA and COL1A1	[48]
	piR013306	Upregulated in HCC	None	[49]
	piR020498	Upregulated in high‐grade dysplastic nodules and advanced HCC	None	[49]
	piR‐like in liver (LLi) 30552	Upregulated in high‐grade dysplastic nodules and advanced HCC	None	[49]
	piR‐Hep1	Upregulated in HCC	None	[50]

## 2. Molecular Mechanisms and Potential Targets of ncRNAs in Liver Diseases

### 2.1. lncRNAs in Liver Diseases

#### 2.1.1. lncRNAs in HCC

Previous studies have shown that lncRNAs are associated with the cell cycle. For instance, Yang et al. [51] explored the effects of HCC‐upregulated EZH2‐associated lncRNA (lncRNA HEIH) in vitro and in vivo via silencing and overexpression experiments. They reported that in hepatitis B virus (HBV)–related HCC, lncRNA HEIH is linked to tumor recurrence and serves as an independent prognostic marker for survival. They also noted that lncRNA HEIH plays a significant role in G0/G1 arrest by upregulating Enhancer of zeste homolog 2 expression [51]. Similarly, lncRNAs associated with liver regeneration (LALR1) have been linked to liver regeneration and proliferation [52, 53]. In vitro, lncRNA LALR1 increased hepatocyte proliferation by promoting cell cycle progression. It also stimulated the growth of mice hepatocytes and the progression of the cell cycle by facilitating cyclin D1 expression and activating the Wnt/β‐catenin signaling pathway via Axin1 suppression [52]. Additionally, in vitro and in vivo experiments have shown that knockdown of lincRNA00974 inhibits HCC cell growth and invasion by causing cell cycle arrest [27].

Several studies have shown that the oncofetal lncRNA plasmacytoma variant translocation 1 (PVT1) is associated with HCC development. The expression of lncRNA PVT1 is upregulated in human HCC tissue samples [28, 54, 55]. It exerts a pro‐tumorigenic effect by enhancing the stem cell‐like properties and proliferation of HCC cells by stabilizing the nucleolar protein NOP2, enhancing nucleolar activity, and promoting cell proliferation by regulating the cell cycle [55]. PVT1 promotes HCC progression by modulating miRNA‐214 [28].

Another lncRNA, termed upregulated in HCC (URHC), is consistently URHC cell lines and tissues. URHC knockdown inhibits HCC cell proliferation and enhances apoptosis by activating sterile alpha motif and the leucine zipper‐containing kinase ZAK via the extracellular signal‐regulated kinase (ERK)/mitogen‐activated protein kinase (MAPK) pathway [31]. Furthermore, the lncRNA metallothionein 1D pseudogene (MT1DP) is inhibited by Yes‐associated protein (YAP) and runt‐related transcription factor 2, both of which are required for liver tumorigenesis [56]. lincRNA‐p21, another lncRNA reduced in HCC, has been shown to inhibit tumor invasion when upregulated by modulating Notch signaling‐induced epithelial–mesenchymal transition [30].

#### 2.1.2. lncRNAs in Liver Fibrosis

lncRNAs play significant roles in the development of liver fibrosis. Shen et al. [57] reported that the lncRNA highly upregulated in liver cancer (HULC), which is abundantly expressed in liver cancer, is also involved in the development of liver fibrosis in NAFLD. High lncRNA HULC levels have also been observed in liver tissues from rats with NAFLD. Additionally, a study using a rat model of NAFLD showed that the inhibition of HULC attenuated MAPK signaling, ameliorated liver fibrosis, and reduced hepatocyte apoptosis [57].

He et al. [58] reported that the lncRNA maternally expressed gene 3 (MEG3) is expressed at considerably lower levels in human fibrotic livers than in healthy livers. Additionally, its expression is downregulated in transforming growth factor (TGF)‐β1‐induced LX‐2 HSC cells [58]. Moreover, lncRNA‐PVT1 expression is upregulated in activated HSCs and fibrotic liver tissue, and its depletion suppresses HSC activation and collagen accumulation in vivo [59]. Furthermore, TGF‐β‐mediated activation of the lncRNA activated by TGF‐β (ATB) is elevated in fibrotic liver tissues, and its knockdown attenuates catenin expression via the upregulation of endogenous miRNA‐200a expression [60].

Some studies have shown that lncRNAs inhibit liver fibrosis. For instance, Zheng et al. [61] observed reduced expression of lincRNA‐p21 in human cirrhotic liver tissue and a mouse model of liver fibrosis. In vitro experiments have also shown that lincRNA‐p21 overexpression inhibits the activation of HSCs and upregulates p21 expression at both the mRNA and protein levels, suggesting that lincRNA‐p21 inhibits cell cycle progression and proliferation in primary HSCs [29]. In contrast, Tu et al. [29] reported a significant increase in hepatocyte lincRNA‐p21 levels during liver fibrosis. Specifically, they found that this elevated lincRNA‐p21 expression in hepatocytes was associated with the downregulation of miRNA‐30 expression, which normally represses TGF signaling via Krueppel‐like factor 1. Moreover, lncRNA‐p21 enhances hepatocyte apoptosis and suppresses hepatocyte proliferation, both in vitro and in vivo [29]. The lincRNA‐p21 expression‐ and function‐related discrepancies between the results of Zheng et al. [61] and Tu et al. [29] can be attributed to several key factors, primarily tissue‐specific expression and differences in experimental models [62, 63]. Transposable elements (TEs) and the lncRNA core promoter that might affect the lncRNA regulatory network are responsible for tissue‐specific lncRNA regulation [64–66].

### 2.2. circRNAs in Liver Diseases

#### 2.2.1. circRNAs in HCC

circRNAs are linked to the progression and development of HCC owing to their ability to target specific miRNAs and proteins. Han et al. [32] observed that circRNA‐mitochondrial translation optimization 1 homolog (circMTO1) suppresses HCC cell growth by sponging miRNA‐9 and upregulating the expression of *p21*, a cell cycle‐related gene targeted by miRNA‐9. Similarly, circLARP4 induces cell cycle arrest and senescence by sponging miRNA‐761, thereby suppressing HCC proliferation. Notably, circLARP4 expression is low in patients with HCC [67]. Moreover, HCC tissues and cells show significant dysregulation of circ0078710. Patients with HCC with high circ0078710 expression often show advanced disease stages (tumor‐node‐metastasis [TNM] classification Stages III–IV). Overexpression of circ0078710 in HCC upregulates histone deacetylase and cyclin‐dependent kinase 2 levels via sponging of miRNA‐31 [37]. Additionally, circBIRC6 is markedly overexpressed in HCC tissues and is associated with overall patient survival. Inhibiting circBIRC6 expression by targeting the miRNA‐3918/B‐cell lymphoma 2 axis induces apoptosis and reduces HCC cell proliferation [35].

The tissue and serum levels of circZEB1.33 are associated with the TNM stages and prognosis of patients with HCC, as circZEB1.33 stimulates HCC cell growth by targeting miRNA‐200a‐3p/cyclin‐dependent kinase 6 [68]. Furthermore, circMTO1 (circ0007874/circ104135) expression is significantly downregulated in HCC tissues, and patients with HCC with low circMTO1 expression have relatively short survival times [69]. Silencing circMTO1 results in the downregulation of *p21* expression, thereby promoting HCC cell growth and invasion. The tumor‐suppressive function of circMTO1 in the development of HCC is demonstrated by its ability to inhibit HCC growth by acting as a sponge for miRNA‐9 and upregulating *p21* expression [69]. In HCC cells, circ100338 enhances invasion by directly interacting with and sponging miRNA‐141‐3p [34]. Additionally, circMTO1 inhibits HCC progression via the miRNA‐541‐5p/Zic Family Member 1 axis by modulating epithelial‐to‐mesenchymal transition and the Wnt/β‐catenin signaling pathway [33].

circRNAs are also implicated in HCC angiogenesis. For example, circ0000092 levels are significantly increased in HCC tissues and cell lines, where they promote angiogenesis by targeting miRNA‐338‐3p and upregulating the expression of hematopoietic‐ and neurologic‐expressed sequence 1, vascular endothelial growth factor, and matrix metallopeptidase 9. Depletion of circ0000092 inhibits HCC cell angiogenesis, growth, migration, and invasion [70]. Moreover, circRNA ephrin type‐B receptor 4 (circEPHB4 and circ0001730) represses hypoxia‐inducible factor‐1α expression and exerts antitumor effects by inhibiting tumorigenesis and metastasis [71]. Similarly, silencing circ0046600 inhibits the expression of hypoxia‐inducible factor‐1α and N‐cadherin in HepG2 and sk‐Heo‐1 cells [72]. HCC tissues exhibit higher expression levels of circRNA cerebellar degeneration‐related protein 1 antisense (circCDR1as and ciRS‐7) than normal adjacent tissues [36]. Silencing circCDR1as inhibits HCC cell invasion and proliferation, while HCC progression is modulated by the circCDR1 as/miRNA‐1287/rapidly accelerated fibrosarcoma (Raf)‐1 axis via the MAPK/ERK pathway [36]. Additionally, circ0008285 knockdown inhibits tumor invasion, migration, and proliferation via the miRNA‐384/ribonucleotide reductase subunit M2 axis [73]. circRNA G protein‐coupled receptor 137B (circGPR137B and circ0017114) has also been shown to suppress HCC cell growth and invasion by targeting miRNA‐4739. The m6A demethylase fat mass and obesity‐associated protein (FTO) is regulated by the circGPR137B/miRNA‐4739 axis, and its expression is indicative of favorable prognosis in HCC [74]. Furthermore, circ0004018 inhibits HCC cell migration and proliferation via the phosphoinositide 3‐kinase/protein kinase B (AKT) signaling pathways [75]. Additionally, circ0004018 expression is lower in HCC tissues, where it targets miRNA‐626/Dickkopf 3 (DKK3) [43].

#### 2.2.2. circRNAs in Liver Fibrosis

circRNAs are ncRNAs that form a covalently closed loop structure, resulting in stability and resistance to exonuclease breakdown [76]. circRNAs are implicated in liver pathology owing to their ability to interact with specific miRNAs and proteins through miRNA sponging and signaling pathway modification [76, 77]. These mechanisms enable circRNAs to play a role in proliferation, apoptosis, differentiation, and inflammation, all of which are important in liver disease development and progression [78]. circRNAs may also influence signaling pathways, including binding to RNA‐binding proteins, acting as protein scaffolds, and modulating gene transcription [79]. circRNAs have been proposed as promising biomarkers and therapeutic targets in diseases, such as chronic hepatitis, liver fibrosis, and HCC because of their diverse functions.

Increasing evidence suggests that circRNAs affect HSC activation, a key process in liver fibrosis. For example, circ0070963 represses HSC activation via miRNA‐223‐3p sponging [80]. circRNA mitochondrial tRNA translation optimization 1 (circMTO1) suppresses HSC activation through both the miRNA‐17‐5p/Smad7 and miRNA‐181b‐5p/phosphatase and tensin homolog (PTEN) axes, displaying predictive value in chronic hepatitis B (CHB)–related fibrosis [81, 82]. The circRNAs Pleckstrin and Sec7 domain‐containing (CircPSD3) and circRNA ubiquitin‐conjugating enzyme E2 K (circUbe2k and circ0009154) modulate Smad7 and TGF‐β2 signaling via miRNA‐92b‐3p and miRNA‐149‐5p, respectively [83, 84]. circRNA F‐box and tryptophan‐aspartic acid repeat domain‐containing 4 (CircFBXW4), downregulated during fibrosis, and attenuates fibrogenic injury upon overexpression, suggesting its potential as a biomarker [38]. Moreover, circRNA death‐inducer obliterator 1 (circDIDO1 and circ0061137) and circ608 exhibit antifibrotic effects through the PTEN/AKT and miRNA‐222 pathways [39, 85].

#### 2.2.3. circRNAs in Intrahepatic Cholangiocarcinoma (iCCA)

iCCA is a rare and aggressive intrahepatic bile duct epithelial cell‐derived primary liver cancer distinct from HCC, which arises from hepatocytes, the main liver cells [86, 87]. While both iCCA and HCC are liver cancers, their cellular origins, risk factors, clinical presentations, treatment strategies, and prognoses differ significantly, thereby allowing for accurate differentiation crucial for patient management. iCCA often presents at an advanced stage, contributing to its poor prognosis [88, 89]. iCCA is the second most common primary liver tumor after HCC. Previous studies have shown that iCCA accounts for 10%–15% of all primary liver cancer cases [90]. Over recent decades, the incidence and mortality of iCCA have significantly increased globally [91]. Although iCCA is clinically and biologically distinct from HCC, iCCA is considered a chronic liver disease. Similar to HCC and liver fibrosis, iCCA develops within a hepatic microenvironment characterized by chronic inflammation, fibrosis, and intrahepatic tumor growth [92].

Accumulating evidence suggests that dysregulated ncRNAs act as molecular regulators in multiple liver pathologies. Therefore, ncRNA‐mediated regulatory networks provide mechanistic insight into iCCA cholangiocarcinogenesis, as well as the molecular mechanisms underlying liver fibrosis and primary liver cancers [93–95]. Several studies have reported the miRNA‐regulated migration, invasion, and chemoresistance in cholangiocarcinogenesis, HCC, and liver fibrosis [96–98]. Dysregulated lncRNAs promote the development of cholangiocarcinogenesis via the PI3K/AKT signaling pathway [99]. The lncRNA LOC102551149/miRNA‐23a‐5p axis regulates hepatic fibrosis via the PTEN/PI3K/Akt/mTOR pathway [100].

circRNAs play a role in iCCA development. circSMARCA5, a tumor suppressor, has been extensively studied owing to its role in the development of different malignancies, including gastric cancer, prostate cancer, and HCC [101–103]. Reduced circular RNA SWI/SNF‐related, matrix‐associated, actin‐dependent regulator of chromatin subfamily A member 5 (circSMARCA5) expression is inversely correlated with overall survival and advanced TNM stage in iCCA tissues [40]. Notably, circular RNA actinin alpha 4 (circACTN4 and circ0050900) is activated in iCCA, where it acts as a sponge for miRNA‐424‐5p, which is believed to target YAP‐1 in the cytoplasm, and recruits Y‐box binding protein 1 to initiate *FZD7* transcription [45]. Therefore, circACTN4 functions as a signaling hub that enables the coordinated activation of the Hippo and Wnt signaling pathways in iCCA [45]. Furthermore, circ0001649 is associated with cell proliferation, migration, and invasion in cholangiocarcinoma (CCA) and HCC [41, 42]. circ0005230 expression is associated with increased tumor size, positive lymph node invasion, and advanced TNM stage in patients with CCA. Additionally, circ0005230 expression markedly enhances CCA cell proliferation and metastatic properties while suppressing apoptosis by sponging miRNA‐1238 and miRNA‐1299 in vitro [44]. In CCA tissues and cell lines, low expression of circ0001649 may be related to an aggressive phenotype [41]. Xu et al. [104] reported that circular RNA 3‐hydroxy‐3‐methylglutaryl‐CoA synthase 1 (cicHMGCS1‐016 and circ0008621) expression in patients with iCCA is related to their prognosis and the likelihood of tumor recurrence, with increased expression promoting iCCA development both in vivo and in vitro. circ0000284 expression is elevated in CCA cells and exosomes from CCA [105, 106]. Recent studies have demonstrated that circRNA nuclear factor I B (circNFIB and circ0086376) expression is markedly downregulated in human iCCA tissues and suppresses iCCA cell proliferation and metastasis by modulating the MAPK1/ERK signaling pathway [46]. Furthermore, circ0059961 expression is significantly downregulated in CCA tissues and prevents CCA proliferation and metastasis by altering the miRNA‐629‐5p/secreted frizzled‐related protein 2 axis [47].

### 2.3. piRNAs in Liver Diseases

piRNAs regulate transcriptional and posttranscriptional gene silencing, thereby playing a key role in maintaining DNA integrity. piRNAs also regulate gene expression by altering chromatin and regulating DNA methylation and repressive histone marks [107–109]. Additionally, piRNAs can epigenetically silence TEs [110]. The direct binding of PIWI proteins to piRNAs plays a crucial role in piRNA biogenesis and functions [111]. PIWI proteins also play multiple roles in cancer, including regulation of cell proliferation, migration, invasion, and genomic integrity, rendering them potential diagnostic and prognostic biomarkers for cancer [112].

PIWI proteins are abnormally expressed in HCC, and their depletion inhibits tumor invasion and metastasis [113, 114]. Although PIWI protein dysregulation in HCC has been reported, studies on the specific roles of piRNAs in HCC are limited. However, some studies have highlighted the involvement of piRNA in HCC progression. For instance, Rizzo et al. [49] identified several piRNAs that are expressed during the early stages of HCC, such as piR013306, which is URHC. Additionally, piR020498 and piR‐like in liver (LLi) 30552 are upregulated in high‐grade dysplastic nodules and advanced HCC [49]. Furthermore, Law et al. [50] demonstrated that relative to the corresponding adjacent nontumor liver tissues, HCC tissues exhibit piR‐Hep1 overexpression. Additionally, piR‐Hep1 silencing decreases AKT phosphorylation, which inhibits HCC cell viability and invasive capacity [50]. Moreover, piR823 has been implicated in the production of ECM components, specifically α‐smooth muscle actin (α‐SMA) and collagen type I alpha 1 (COL1A1), ultimately leading to cirrhosis [48].

piRNA dysregulation in noncancerous liver diseases has been recently described. piRNA expression was altered in a NAFLD model, suggesting its potential involvement in disease progression and fibrosis [115, 116]. PIWI‐interacting RNA‐23210 is associated with acetaminophen‐induced hepatotoxicity by protecting against liver injury through targeting hepatocyte nuclear factor 4 alpha and hepatocyte nuclear factor 1 alpha [117]. Although current findings suggest potential roles for piRNAs in noncancerous liver diseases, most evidence remains preliminary and requires validation in large human cohorts. The exact biological mechanisms through which piRNAs influence disease progression, whether via gene regulation, epigenetic modulation, or interactions with other RNA molecules [118, 119], remain largely elusive. Therefore, despite the growing interest in piRNAs, their role in liver pathology still remains incompletely understood, underscoring a critical gap in the literature and the need for more targeted investigations.

### 2.4. miRNAs in Liver Diseases

Tumor‐suppressor and oncogenic miRNA dysregulation enables uncontrolled cell division by modulating signaling pathways essential for cell survival, proliferation, metabolic reprogramming, and metastasis. miRNA‐122 expression is significantly reduced in HCC [120]. In addition, miRNA‐122 suppresses Toll‐like receptor 4 (TLR4) expression, thereby inhibiting the activities of PI3K, AKT, and nuclear factor kappa‐light‐chain‐enhancer of activated B (NF‐κB) cells, which in turn reduces inflammatory cytokine production [121]. miRNA‐26a also promotes HCC apoptosis by directly targeting Janus kinase (JAK) 1 expression, thereby regulating the JAK/STAT3 survival pathway [122]. miRNA‐21 significantly increases HCC cell migration and invasive capabilities by targeting Kruppel‐like factor 5 [123]. However, recent studies have reported that total or hepatocyte‐specific deficiency of miRNA‐21 promotes HCC development, contrary to its expected oncogenic role [124]. These conflicting results highlight the need for further studies on the role of miRNA‐21 in the progression of HCC.

Several liver‐enriched miRNAs play important roles in hepatic homeostasis and the pathology of liver diseases. Among these, miRNA‐122, accounting for approximately 70% of total hepatic miRNAs, is a crucial regulator of lipid metabolism, inflammation, and hepatocarcinogenesis in HCC and other liver diseases, such as NAFLD [121, 125]. Dysregulation of miRNA‐122 is involved in the development of liver fibrosis and HCC through lncRNAs and circRNAs [126]. In HCC, lncRNA RPPH1 functions as a molecular sponge for miRNA‐122, which regulates Wnt1/β‐catenin signaling [127]. The regulatory effects of circ0055054 and miRNA‐122‐5p on Mucin 1 expression modulates the proliferation, migration, and invasion of HCC cells [128]. Similarly, miRNA‐34a, a critical mediator of p53‐dependent signaling, is upregulated in liver diseases, such as NAFLD, HCC, and liver fibrosis [129–131]. miRNA‐34a is also involved in apoptosis, tumor suppression, cell cycle regulation, and epithelial–mesenchymal transition in concert with lncRNAs and circRNAs that act as molecular sponges in liver diseases [132, 133].

Metabolic dysfunction–associated steatotic liver disease (MASLD), formerly NAFLD, is a major global health concern, affecting over 1 billion adults worldwide, and represents a disease spectrum ranging from simple steatosis to metabolic dysfunction‐associated steatohepatitis (formerly nonalcoholic steatohepatitis [NASH]), cirrhosis, and ultimately HCC [134]. The levels of miRNA‐122, the predominantly expressed miRNA in the liver, increase before those of serum alanine aminotransferase and reflect the histological and molecular processes occurring in the liver [135, 136]. miRNA‐34a is also upregulated in NAFLD and induces apoptosis, cell‐cycle arrest, and cell senescence by regulating the expression of several genes, including B‐cell lymphoma 2, the MYC proto‐oncogene, basic helix–loop–helix transcription factor (MYC), and Sirtuin 1 [135]. Reduced miRNA‐145a‐5p expression facilitates the transition from steatosis to NASH through upregulation of nuclear receptor subfamily 4 group A member 2 [137].

## 3. ncRNAs as Biomarkers and Targets in Liver Diseases

**Figure 1:**
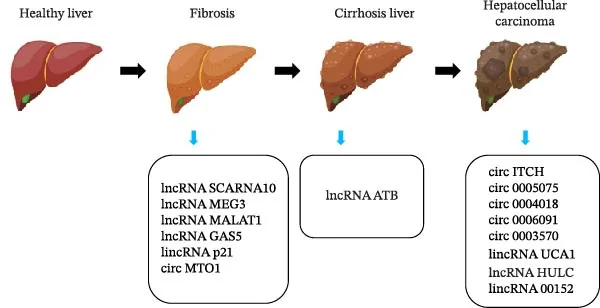
Potential biomarkers for liver disorders, such as fibrosis, cirrhosis, and hepatocellular carcinoma, have been identified in serum and plasma. These biomarkers include various noncoding RNAs (ncRNAs) and circular RNAs (circRNAs).

ncRNAs have been identified in human biofluids and in culture supernatants in vitro [138]. Some circulating lncRNAs have been reported as potential biomarkers for liver diseases [139, 140]. In Section 3, we summarize key lncRNAs and circRNAs identified in serum and plasma that show promise as biomarkers for liver diseases (Figure 1).

Recent studies have significantly advanced machine learning (ML)–based biomarker discovery and diagnostic panel development. ML approaches are becoming an important part of ncRNA–based research, particularly in identifying novel disease‐associated lncRNAs and clinically relevant ncRNA biomarkers [141]. Recent studies have used ML models to integrate ncRNA data into clinical workflows for cancer diagnostics [141, 142]. Several studies have developed novel prognostic scoring systems for the accurate prognosis of patients with HCC by integrating lncRNA and ML [143–145]. Indeed, artificial intelligence, ML, and deep learning have been employed to create robust in silico predictive models for the application of precision medicine in liver diseases [146].

Antisense oligonucleotides (ASOs) are synthetic single‐stranded nucleic acids designed to bind to specific RNA sequences, leading to RNA degradation or inhibition of their function [147, 148]. This approach is particularly promising for targeting lncRNAs in cancer treatment [149, 150]. The development of ASOs targeting the oncogenic lncRNA HOX transcript antisense RNA and metastasis‐associated lung adenocarcinoma transcript 1, both implicated in the progression and metastasis of various cancers, represents a promising therapeutic strategy in RNA–based oncology [151]. A recent study reported the first‐in‐human clinical trial for ASOs targeting the lncRNA taurine upregulated gene 1 (TUG1ASO)―an antisense oligonucleotide targeting the lncRNA TUG1 in recurrent glioblastoma―to assess safety, tolerability, and efficacy, marking a significant step in lncRNA‐targeted therapy [152].

In addition, siRNAs and clustered regularly interspaced short palindromic repeat (CRISPR)–based targeting of ncRNAs have been applied in preclinical or clinical trials [142]. siRNA techniques are a well‐established approach for sequence‐specific silencing of pathogenic ncRNAs and have shown promising efficacy in preclinical liver disease models. N‐acetylgalactosamine‐siRNA has been applied to silence lncRNAs, which ameliorates liver fibrosis caused by *Clonorchis sinensis* [153]. The liver is one of the most accessible organs for RNA–based therapeutics, enabling siRNA–based approaches [154]. However, the use of siRNAs against specific ncRNAs, such as lncRNAs or circRNAs, is still at the preclinical stage [142]. CRISPR‐associated nuclease 9‐mediated genome editing and CRISPR interference have been developed to modulate ncRNA expression at the transcriptional level [155, 156]. However, these approaches have remained at the preclinical stage owing to insufficient delivery efficiency, off‐target effects, and concerns about long‐term safety [157].

Quantitative real‐time polymerase chain reaction, microarrays, and next‐generation sequencing have been used to detect ncRNAs with potential as diagnostic and prognostic biomarkers [158, 159]. In addition, ncRNA expression profiles are regulated by a variety of preanalytical and biological parameters, including sample type (tissue, plasma, serum, or exosomal fractions), storage conditions, and patient characteristics [160]. miRNAs are easily detectable in liquid biopsies because they are secreted into the circulation by cells [161]. In contrast, lncRNAs are difficult to detect due to their low levels in circulation [162]. Furthermore, many candidate ncRNA biomarkers have been evaluated in relatively small and homogeneous patient cohorts [155]. Therefore, large‐scale and multicenter validation studies across diverse populations are necessary to assess the diagnostic performance and reproducibility of these biomarkers in clinical applications.

### 3.1. circRNAs as Biomarkers

circRNAs have emerged as therapeutic targets and diagnostic biomarkers for HCC. Several studies have identified specific circRNAs, such as hsa_circ0005075 and hsa_circ0004018, as potential diagnostic biomarkers for HCC [163–165]. Additionally, low expression of circMTO1 has been correlated with poor prognosis in patients with HCC [32]. Consistently, serum circMTO1 levels have been inversely correlated with fibrosis stage and histological activity index score [81]. Furthermore, circRNA itchy E3 ubiquitin protein ligase (circITCH) levels are elevated in the plasma of patients with HCC, especially in those with Child‐Pugh class C cirrhosis [166]. circITCH expression and the levels of liver enzymes, such as aspartate aminotransferase and alanine aminotransferase (indicators of hepatocyte damage), are positively correlated. The receiver operating characteristic (ROC) curve for plasma circITCH levels had an area under the ROC value of 0.661 (95% confidence interval: 0.5433–0.778), with specificity and sensitivity being 65% and 70%, respectively, suggesting that circITCH has potential diagnostic value in HCC [166].

Furthermore, serum hsa_circ0006091 levels in patients with HCC were significantly decreased 1 and 7 days after surgery [167]. In HCC tissues, hsa_circ0005986 expression is downregulated, while high levels of hsa_circ0005986 are associated with enhanced survival [168]. Moreover, hsa_circ0003570 expression gradually decreases with disease progression from chronic hepatitis to liver cirrhosis to HCC [169]. Jang et al. [169] showed that hsa_circ0003570 is associated with survival and disease progression in patients with HCC, indicating that it may be a useful prognostic biomarker for HCC.

### 3.2. lncRNAs as Biomarkers

The potential of lncRNAs as biomarkers in liver diseases, particularly HCC, has gained significant attention, and numerous pharmaceutical companies have developed agents targeting lncRNAs [170, 171]. The functions of lncRNAs can be regulated by siRNAs that target specific lncRNAs [68]. Moreover, ASOs can be modified for condition‐specific lncRNA targeting [172]. Several studies have revealed that lncRNAs can be employed as biomarkers for HCC diagnosis. One study reported that when lncRNAs are combined with serum alpha‐fetoprotein levels, the sensitivity associated with their use as biomarkers increased to 100%, suggesting promising utility as biomarkers for HCC [173]. Furthermore, lncRNAs, such as lncRNA HULC, which are HULC, are more frequently detected in plasma samples from patients with HCC than in those from healthy controls. Patients with higher Edmondson grades or HBV‐positive status show higher plasma lncRNA HULC detection rates [174].

In a previous study, patients with CHB exhibited low serum lincRNA‐p21 levels, which was found to be negatively correlated with liver fibrosis stage, demonstrating the diagnostic utility of lincRNA‐p21 for HCC [175]. Additionally, serum lincRNA‐p21 levels were negatively associated with the liver fibrosis markers α‐SMA and COL1A1. ROC analysis suggested that blood lincRNA‐p21 levels are a promising diagnostic biomarker for liver fibrosis [175]. Another study demonstrated that serum small Cajal body‐specific RNA 10 levels are relatively high in patients with advanced liver fibrosis [176]. Moreover, patients with HCC have significantly higher serum lincRNA00152 and UCA1 levels than patients with hepatic cirrhosis and healthy controls [177].

According to Chen et al. [178], patients with CHB exhibit low serum lncRNA MEG3 levels, which is negatively related to the degree of liver fibrosis. Furthermore, serum samples from patients with HBV‐associated liver fibrosis show significantly higher levels of lncRNA metastasis‐associated lung adenocarcinoma transcript 1 (MALAT1) than those from healthy controls. MALAT1 enhances liver fibrosis by targeting miRNA‐181 and activating the Toll‐like receptor 4‐NF‐κB pathway [179]. Moreover, patients with advanced fibrosis exhibit higher plasma lncRNA growth arrest specific 5 (GAS5) levels than their healthy counterparts. However, plasma lncRNA GAS5 levels are lower in patients with cirrhosis than in those with fibrosis. Compared with healthy controls, patients with HBV‐related cirrhosis exhibit significantly increased plasma lncRNA‐ATB levels [180].

## 4. Conclusions

In liver diseases and injury, ncRNAs regulate multiple critical aspects of liver physiology and pathology. In addition, they are essential to the pathophysiology and metastasis of HCC and liver fibrosis. ncRNA‐mediated regulatory mechanisms involve posttranscription repression, miRNA sponging, signaling pathway modulation, and fibrosis‐related gene regulation in liver disease (Table 2). Although recent studies have reported the application of ncRNAs in the development of therapeutic agents, this field remains at an early stage of translational and clinical development [183, 184]. Therefore, further studies should focus on combining ncRNA expression profiles with extensive clinical information (such as patient demographics, disease stage, and response to treatment), as well as imaging modalities, such as computed tomography (CT) and magnetic resonance imaging (MRI), for developing robust diagnostic panels and enabling individualized treatment options [185, 186]. In summary, integration of ncRNA data with clinical and imaging frameworks is crucial to advancing precision medicine for liver diseases, particularly HCC and liver fibrosis.

**Table 2:** Common ncRNA‐mediated regulatory mechanisms in liver diseases.

ncRNA class	Common mechanism	Representative examples	Liver disease context	Reference
miRNAs	Posttranscriptional repression	miRNA‐214, miRNA‐30	Fibrosis, HCC	[181, 182]
lncRNAs	miRNA sponging	PVT1–miRNA‐214, lincRNA‐p21–miRNA‐30	Fibrosis, HCC	[28, 29]
circRNAs	miRNA sponging	circMTO1–miRNA‐9, circACTN4–miRNA‐424‐5p	HCC, iCCA	[32, 45]
circRNAs	Signaling pathway modulation	circNFIB–MAPK/ERK	iCCA	[46]
piRNAs	Fibrosis‐related gene regulation	piR823–α‐SMA/COL1A1	Cirrhosis, HCC	[48]

Future research should focus on integrative multi‐omics approaches that combine ncRNA profiles with mRNA expression, proteomic, and epigenomic data to identify robust diagnostic panels and facilitate the development of combination therapies targeting multiple ncRNA pathways. Furthermore, integrating ncRNA signatures with comprehensive clinical information and imaging data, including CT and MRI, will be essential for improving diagnostic accuracy and predicting treatment responses. Overall, integrative multiomics strategies will be critical for advancing precision medicine in liver diseases and translating ncRNA research into clinically useful tools.

**Nomenclature**
α‐SMA: α‐Smooth muscle actin
circRNA: Circular RNA
COL1A1: Collagen type I alpha
ERK: Extracellular signal‐regulated kinase
FBXW4: F‐box and WD40 domain‐containing protein 4
HCC: Hepatocellular carcinoma
iCCA: Intrahepatic cholangiocarcinoma
lncRNA: Long noncoding RNA
MAPK: Mitogen‐activated protein kinases
MTO1: Mitochondrial translation optimization 1 homolog
piRNA: PIWI‐interacting RNAs
SFRP: Secreted frizzled‐related protein
URHC: Upregulated in HCC.

## Author Contributions

Eun Jung Sohn and Sae‐Ock Oh designed the project, performed experiments, and wrote the manuscript. Eun Jung Sohn conducted data and visualization analysis.

## Funding

This work was supported by the National Research Foundation of Korea and funded by the Ministry of Education (Grant NRF‐2022R1A5A2027161) and by the Medical Research Center (MRC) Program (Grant NRF‐2022R1A5A2027161) funded by the Ministry of Science and ICT (MSIT).

## Ethics Statement

The authors have nothing to report.

## Conflicts of Interest

The authors declare no conflicts of interest.

## Data Availability

The data will be made available upon request.
